# Identifying how tobacco industry-targeted communities perceive California’s tobacco endgame initiative

**DOI:** 10.1186/s13011-025-00674-6

**Published:** 2025-10-01

**Authors:** Rachael A. Record, Katya Azzam, Mary Margaret Gonzales, Lydia H. Greiner, Georg E. Matt

**Affiliations:** 1 School of Communication, San Diego State University, San Diego, CA USA; 2 Department of Psychology, San Diego State University, San Diego, CA USA; 3 5500 Campanile Dr, San Diego, CA 92182 USA

**Keywords:** Tobacco endgame, Underserved communities, Qualitative, Big tobacco, Tobacco prevention

## Abstract

The Tobacco Endgame is a global initiative that seeks to implement tobacco prevention policies to collectively end the commercial tobacco epidemic and eliminate tobacco-related health disparities around the world. California seeks to be among the first regions to effectively implement such an initiative, with a statewide Tobacco Endgame goal of 2035. Given the tobacco industry’s manipulation marketing tactics that perpetuated tobacco-related disparities among select communities, California’s Tobacco Endgame prioritizes these communities (i.e., African American, Hispanic, Asian American/Pacific Islander, and LGBTQ+). To effectively support underserved communities as they prepare for Tobacco Endgame-related policies, research is needed to understand their perspectives and priorities. Through a qualitative approach, this study explored the present awareness and perspectives of California’s priority communities regarding the Tobacco Endgame. Following virtual focus groups (*n* = 19), analyses revealed four shared themes that appeared in all four community groups (i.e., health implications, addiction, post-implementation challenges, children and youth) as well as at least two unique themes that emerged within each of the four priority communities. Findings highlight community-specific concerns, including the prioritization of flavored products, an emphasis on the environmental impact, and the need to protect communities from black market sales, that can inform targeted communication and education outreach. In addition, shared concerns for health, enforcement challenges, and children can inform communication and outreach approaches for a statewide awareness campaign that could resonate broadly.

After more than 60 years of dedicated tobacco-related research, education efforts, tobacco control policies, and millions of dollars spent, tobacco control advocates, policymakers, and researchers are pivoting to a final phase of tobacco control planning: the Tobacco Endgame [1–3]. Broadly defined, the Tobacco Endgame encompasses work that moves “beyond tobacco control (which assumes the continued presence of tobacco as a common, widely available, ordinary consumer product) toward a tobacco-free future wherein commercial tobacco products would be phased out or their use and availability significantly restricted” (p. 594) [8]. In other words, the Tobacco Endgame envisions a world free of the burden of tobacco-related death and disease. Given California’s successful history of reducing the smoking rate, implementing tobacco-free policies, and regulating the sale of tobacco products [4–6], many advocates and researchers believe California to be uniquely positioned to be the first in the U.S. to achieve a statewide Tobacco Endgame [9]. According to the California Tobacco Endgame Policy Initiative, co-published by the Public Health Law Center and the American Lung Association, California’s tobacco prevention advocates believe they can achieve the Tobacco Endgame by 2035 [7].

California’s Tobacco Endgame initiative seeks to eradicate the tobacco industry’s influence and harm through building a statewide movement that prepares and transitions communities, especially among populations that the tobacco industry has historically targeted, to (a) end the commercial tobacco epidemic, (b) protect public health, and (c) eliminate tobacco-related health disparities [7]. Conceptually, the Tobacco Endgame envisions a world free of the burden of tobacco-related death and disease. Operationally, the Tobacco Endgame encompasses initiatives designed to permanently change the structural, political, and social dynamics that sustain the commercial tobacco epidemic [10]. To date, and coupled with the implementation of comprehensive environmental smokefree policies [1], most Tobacco Endgame initiatives include policies centered on limiting tobacco retail sales [11–13], phasing out the legal age of sale [14], and/or prohibiting tobacco sales altogether [15, 16].

Across California, communities will have varying readiness levels for different aspects of the Tobacco Endgame initiative. Communities with the highest smoking rate, highest density of retail shops, and fewest comprehensive tobacco-free policies will be among the highest priority communities. These populations are more commonly, but not exclusively, racial and ethnically underserved minorities and members of the LGBTQ + community of lower socio-economic status [7]. To develop Tobacco Endgame strategies for the identified priority populations, one must first understand the history of Big Tobacco’s influence on these communities.

The commercial tobacco epidemic is built on the structural racism that persists in the U.S., making commercial tobacco use “a fundamental health equity and social justice issue” (p. 5) [7]. As outlined in the Endgame Policy Platform, structural racism, which is embedded into our laws, practices, systems, and cultural and societal norms [17], has resulted in the systemic oppression of communities of color which has led to poorer health outcomes. Private corporations, including Big Tobacco, have historically exploited these structural barriers for their own financial gain. For Big Tobacco, this took the form of predatory targeting of communities of color through pervasive and culturally appropriative advertising, lower-priced products, higher retailer density, and intentionally pushing products, such as menthol cigarettes, that are easier to initiate and more addictive [7, 18, 19]. 

Public health evidence has been mounting for decades regarding the systematic injustices surrounding the burden of tobacco-related disease and death for minority and underserved populations. For instance, compared to California’s overall smoking rate, smoking rates are significantly higher among African American and LGBTQ + communities [20, 21], with menthol-flavored products, in particular, to be disproportionately harming both communities [20]. The same report found the rates of tobacco use by California adults are highest among mixed race and African American communities, and rates by youth are highest among Native Hawaiian/Pacific Islander communities [21]. Further, California’s Latino community holds the second-largest demographic of adult smokers, with an estimated 1.1 million tobacco users [20]. This is especially problematic as lung cancer remains the leading cause of cancer death among California’s Latino men and second among its Latino women [22]. Together, these data demonstrate why the African American, Hispanic, Asian American/Pacific Islander (AAPI), and LGBTQ + communities have been identified as priority populations for California’s Tobacco Endgame initiative.

Although the initiatives and policy goals for California’s Tobacco Endgame are appropriate and essential, what is missing is an understanding of how to most effectively garner public support for the effort, particularly among priority populations. Without public support, achieving these goals will not only be more difficult but less likely. As the tobacco industry did not target communities equally, efforts to end the tobacco epidemic cannot be implemented equally across populations. If efforts are expected to best reach and support the identified priority populations, research is needed to provide a foundational understanding of how members of these communities perceive the Tobacco Endgame. To that end, this study sought to explore how priority populations, specifically the African American, Hispanic, AAPI, and LGBTQ + communities, perceive Tobacco Endgame initiatives. Such community-level insights have been shown to provide a framework for guiding messaging needs among diverse target communities [36–38]. The following research questions were qualitatively explored.

RQ1: How are members of tobacco industry-targeted communities perceiving Tobacco Endgame policy efforts?

RQ2: What Tobacco Endgame-related priorities and barriers exist for members of tobacco industry-targeted communities?

## Method

### Procedures & participants

To explore these research questions, focus groups were employed to capture broad perceptions regarding Tobacco Endgame policies, including perceived barriers and benefits of enactment. Participant recruitment occurred virtually throughout California during fall 2022. An application was submitted for review to a university institutional review board, who deemed the project exempt from review due to the educational nature. To be eligible, individuals had to be 18 years old or older, live in California, and identify as a member of the African American, AAPI, Hispanic, or LGBTQ + community. To confirm eligibility and allow participants to sign-up for a focus group session, flyers were created with a QR code directing readers to a Google Form. The form asked participants to report their age, state residency, sexual orientation, and race/ethnicity. Depending on their answers, eligible participants were directed to available sessions and ineligible participants were thanked for their interest. The flyer was first disseminated on the social media platforms of California’s Thirdhand Smoke Resource Center (THSRC), which has broad community reach and followership across California. Additional recruitment strategies included sharing materials with California priority population resource centers and THSRC community partners to disseminate among their networks.

After eligible participants registered for a session, they were emailed reminders 48 h before their scheduled sessions. On the day of their session, 30 min prior to the start, they were emailed a Qualtrics link to a brief demographic survey. The purpose of this survey was to be able to describe the focus group participants without taking up session time or requiring disclosure of personal details. At the end of the survey, participants were provided with the link to the Zoom room for their session. Table 1 presents a demographic summary of participants.

**Table 1:** Focus group demographic characteristics

	Overall	African American	Hispanic	AAPI	LGBTQ+
N	88	29	19	13	27
Age: M (SD)	27.8(14.4)	21.1(13.0)	32.3(7.9)	35.2(20.2)	31.1(9.5)
Gender: Female	37%	21%	79%	77%	22%
Education: Associate or less	41%	34%	48%	46%	41%
Single family home	35%	14%	74%	39%	30%
Renting home	60%	55%	58%	31%	82%
Employed full or part time	76.2%	62%	79%	85%	85%

### Protocol development

The focus group protocol was iteratively developed with project investigators and research assistants. Once drafted, the protocol was pilot-tested using a research participant recruitment platform called SONA at a large university in Southern California where communication and journalism majors received extra credit in their courses in exchange for participating in the pilot-testing. Following pilot testing, the protocol was finalized for use.

The final protocol first welcomed participants, outlined the expectations of the sessions, and answered any participant questions. Next, participants began by sharing what they already knew about the Tobacco Endgame. For those that didn’t have prior knowledge, they were asked to share what the phrase made them think about. Next, participants were provided California’s definition of the Tobacco Endgame. After sharing the definition, the remainder of the session was dedicated to allowing participants to share their thoughts, perspectives, concerns, and ideas regarding five of the Tobacco Endgame’s key policy areas (see Table 2). These five policy statements summarize the nine policy areas outlined in California’s Tobacco Endgame Policy Platform in lay terms [7]. 

**Table 2:** Summary of california’s tobacco endgame policy priorities

Policy Presented to Focus Group Sessions	
1. End the sale of combustible commercial tobacco products 2. End the sale of flavored tobacco products 3. End the sale of tobacco products that contribute to environmental waste 4. Expand smoke-free policies in California to include all public indoor and outdoor places, and any apartment or condo that has a shared ventilation system^a^ 5. Promote the proper cleaning of homes, vehicles, and other material goods for the removal of the toxic tobacco residue^b^	

Note. ^a^Henceforth referred to as “expand smoke-free policies in California”; ^b^Henceforth referred to as “promote the proper cleaning of homes, vehicles, and other material goods”

### Data analysis

All focus groups were conducted by one of two research assistants, Zoom recorded, and auto-transcribed. Within each community, focus groups were scheduled until the community-assigned research assistant felt no new information was being shared within sessions. Following, one more session was held to confirm saturation within each community. A total of 19 focus groups were conducted. One group had a single participant and was conducted as an interview. Once all focus groups were complete, the researchers began the process of validating the transcripts. To do this, a researcher who did not host the focus group was assigned to watch the recording and read the transcript, correcting any errors, formatting, and grammar. Validated transcripts were uploaded to Nvivo [23] for analysis.

The data were thematically analyzed following Creswell [24] to explore broad themes across communities. To independently explore community perceptions, all focus groups from one community were coded at a time, allowing coders to be fully immersed within each community data set. To establish internal consistency in coding practices among the multiple coders, one community (the African American community) was randomly selected to serve as a training group where two coders served as the primary coder with no secondary coder. For these files, each coder independently reviewed and coded the transcripts, creating their own thematic codebook for the African American community. Coders then met to compare codebooks and discuss discrepancies. Disagreements were resolved by the project PI. Once the community codebook was finalized, both primary coders compared the transcripts to the community codebook, confirming all codes were accurately recorded. Moving forward, the remainder of the communities were independently reviewed by a primary coder and verified by a secondary coder. The same process occurred where the primary coder read through the transcripts, identified an initial set of subthemes, and reviewed with the secondary coder. Following this first review, the project PI resolved disagreements and the community codebook was finalized. The primary coder then read through the transcripts again, confirming the overarching themes and final codes.

## Results

Analyses revealed four shared themes that appeared in all four community groups. In addition, unique themes emerged within each community group. Table 3 presents an overview of all the themes. The following paragraphs will explore each theme further.

**Table 3:** Summary of themes from focus group discussions

Shared Themes in all Groups				
Health Implications Addiction		Post-implementation Challenges Children & Youth		
**Unique Themes**				
**African American**	**Hispanic**		**AAPI**	**LGBTQ+**
Environment Economic Impact Criminalization Risks	Flavored Products Policy Suggestions		Impact on Local Business Infringement Concerns	Environment Economic Impact Individual Rights

### Shared themes in all groups

#### Health implications

The first theme that emerged in all four community groups was the positive implications that one or more of the Tobacco Endgame policies would have on public health (see Table 4 for selected quotes). The benefits were considered for both people who did not smoke and people who did. Regarding people who did not smoke, discussions focused on how Tobacco Endgame policies could result in less secondhand smoke exposure in their communities, better protecting the health of nonsmokers. Similarly, participants also considered how Tobacco Endgame policies would improve the health of people who did smoke by making it more likely that they would quit using tobacco products. Overall, the theme of implications for health captures the positive public health impacts that members of all four community groups recognized, suggesting communication efforts from the Tobacco Endgame initiative should leverage the health benefits for both people who smoke and people who do not. But there was an important other side to this discussion that is captured in the next theme.

**Table 4:** Example quotes from shared themes in all groups

Health Implications	Addiction	Post-implementation Challenges	Children & Youth
“I’m a nonsmoker. I don’t smoke, and I don’t take tobacco either. But then people that smoke outdoors in public places put me at risk. They put me at risk because I have to breathe. And when I’m breathing, I’m taking in the tobacco. And this is really dangerous to me.” – Hispanic, female, response to the policy to *expand smokefree policies in California* “Personally, I am not a smoker but indirectly it could harm me, maybe inhaling the product, so this will also be a health benefit—and as well as to my family” – LGBTQ, male, response to the *policy to end the sale of combustible commercial tobacco products* “The use of tobacco comes with health challenges and brings an increased mortality rate. So when we have such a policy, it will help my family well, and the community.” – African American, male, response to the *policy to end the sale of combustible commercial tobacco products*	“If you stop the sales in California now, people will look for new ways to try to get it. People are just going to look for it in any other state in which it is legal.” – Hispanic, female, response to the policy to *end the sale of combustible commercial tobacco products* “It’s going to be difficult for people with addiction and it’s going to be good in the aspect of health because people will be healthy.” – African American, male, response to the policy to *expand smoke free policies in California*	“I would even push it to the next level, having some of those policies that earmark funds for underserved communities, because a lot of times the usual suspects will get it, which is the big three American Heart, Lung, Cancer. I truly feel that alongside those policies that it should be complemented with earmarked funding for underserved communities.” – AAPI, male, response to request for *any other thoughts* “Eliminating tobacco supply from major companies will encourage black market sales. Well, I think this black market could be even more harmful than what we use, you know? Because the products used to make this, to make cigarettes and all, would be more harmful.” – LGBTQ, male, *response to the policy to end the sale of combustible commercial tobacco products*	“The introduction of tobacco habits starts when in their younger years, and so they have been targeted—and devastatingly—to the point where it does affect them. I work in a school, and I know students who have, unfortunately, even though it is against policy. Obviously, you know, because it’s not supposed to be available to them, but you know they put themselves in danger of being kicked out of the school just for the chance to be able to use it. And so, I mean, just making it inaccessible. It would make a huge difference.” – Hispanic, female, response to the *policy to end the sale of flavored tobacco products* “I stopped smoking some years ago and I’ve been trying to be an advocate to people around me, especially my little brother. That policy, I hope, will make tobacco difficult for his consumption and will also have a nice effect on him and some other folks like him.” – African American, male, response to the *policy to end the sale of flavored tobacco products*

#### Addiction

Participants in all four community groups recognized that tobacco is an addictive product and that protective policies the promote public health do not change the addictive nature of the product or the challenges that users have to navigate on their cessation journeys. For many participants, there was a moral and ethical concern about implementing Tobacco Endgame policies without consideration of addicted users or need for resources to help them quit (see Table 4 for selected quotes). In this same vein, there was also frequent recognition in all four community groups about the lengths that addicted users might go to for their nicotine fix. The addictive nature of tobacco products is clearly recognized across priority populations, suggesting the Tobacco Endgame initiative will need clear communication regarding how addicted users will be affected or supported by any proposed policy change.

#### Post-implementation challenges

The third theme that emerged across all four community groups was a close consideration of the challenges that individuals, communities, and businesses could face following implementation of any Tobacco Endgame policy. The discussed challenges were fairly broad with considerations being given regarding the potential increased use of other products (e.g., marijuana, alcohol), the need for more education on the importance of tobacco control policies, and the need for the provision of cessation services post-policy implementation as part of the policy language (see Table 4 for selected quotes). One of the most vocal concerns across communities was the fear of black markets that sell tobacco products popping up following the implementation of a Tobacco Endgame policy. Altogether, it was clear that the unknown impacts following the implementation of Tobacco Endgame policies was a potential barrier to support of the policies among members of these priority populations. This suggests that the initiative needs to engage in conversations with community partners and leaders to not only reduce perceived worries, but also articulate investments to alleviate these real and valid concerns.

#### Children & youth

The last theme that emerged across all four community groups was the impacts of Tobacco Endgame policies for children and youth. These conversations were almost unanimously framed as the positive impact that each of the Tobacco Endgame policies would have on the next generation (see Table 4 for selected quotes). This included the potential to prevent initiation and to reduce current use before the addiction is too strong, particularly of electronic cigarettes (i.e., vapes). Across all sessions for all communities, youth was consistently at the forefront of participant considerations. The opportunity to create a tobacco-free world for community youth was motivating and almost always perceived as the primary benefit of all Tobacco Endgame policies.

In sum, four themes emerged across all four of the community groups. The themes of health implications, addiction, post-implementation challenges, and children and youth shed light on shared perceptions of Tobacco Endgames policies that resonate across priority populations that have been disproportionately targeted by the tobacco industry. In addition, analyses also revealed themes unique to each of the four community groups.

### Unique themes

#### Unique themes among the African American community

Among focus group participants in the African American community, three additional themes emerged. First, many participants considered the positive impact that the Tobacco Endgame policies would have on the environment to be a priority of the overall initiative, particularly with regard to reduced litter in outdoor environments. The focus group protocol did include one question specifically centered on the environment, but participants in these focus groups brought up the environmental impact well before and after that question. For example, in responding to the policy to *end the sale of combustible commercial tobacco products*, a male participant shared, “The negative effects caused by waste of tobacco on the environment are vast and growing in my neighborhood. This adds unnecessary pressure for our fragile ecosystems. Ending the sale of those products will be beneficial to solving part of the environmental menace.” The ability of Tobacco Endgame policies to promote a cleaner outdoor environment was perceived benefit among African American participants, one that could be leveraged in communication promoting Tobacco Endgame policies.

But distinct barriers for policy support also emerged in the conversations with members of the African American community. First, there were concerns about the potential negative implications that Tobacco Endgame policies could have on the economy, particular regarding the livelihood of small businesses, the loss of tax dollars, and the risky creation of black markets. As an example, a male participant responded to the policy to *end the sale of combustible commercial tobacco products* with the following statement:

Also people will lose jobs. I think tobacco industries are multi- million, if not a multi-billion industry. So I think that lots of taxes will be lost, lots of cash, lots of jobs. So it will be both positive and negative for me.

Many participants recognized the income that tobacco products bring into their communities, particularly for some business owners and local stores. The worry over this loss of income creates a barrier to supporting the Tobacco Endgame that could be overcome with efforts to provide alternative products for sale by these businesses, such as cessation products or healthier products.

Second, there was a fear that these policies would lead to a risk of criminalization among their community members, such as being disproportionally arrested if they were caught smoking. Although none of the policies suggested that criminalizing people who smoke was being considered, the perception was that it could be a risk of increased tobacco control efforts. For example, when responding to the policy about *expanding smokefree policies in California*, one woman made the following comparison:

This kind of policy doesn’t feel good to me and doesn’t feel right in a way. It’s just like you’re seeking a job and your interviewer is asking you about your conviction history. Like a parole staff, it’s always very difficult to be in that space. So, if I have to tell my smoking history to get a house or to buy a car or to have my insurance done. Definitely a lot of people would not stand a chance in owning anything. So, this is going to impact a lot of people, someone like me, negatively in a lot of ways. So, I’m not sure this feels, feels good. It doesn’t feel good to me. And it’s going to affect a lot of people, not just me, my friends, my cousins, everybody that I know.

Given the historical over criminalization of the African American community in the U.S., the risk of criminalization is a deep-rooted fear that could create a real barrier to support for Tobacco Endgame policies—even if the policies do not explicitly criminalize smoking. Considering the ongoing and historical validity of such concerns, it is essential that California’s Tobacco Endgame planners take care in ensuring that proposed policies would not put the African American community at greater risk of criminalization and, most importantly, that efforts are made to articulate exactly why that risk is not present.

#### Unique themes among the Hispanic community

Regarding the focus groups with members of the Hispanic community, two unique themes emerged. The first was the importance of the Tobacco Endgame initiative to end the sale of flavored tobacco products, especially vapes. The focus group protocol did include one question specifically centered on ending the sale of flavored tobacco products, but for these focus groups, participants brought up flavored tobacco products well before and after that question. Overall, participants generally agreed that Tobacco Endgame policies would be an important step in reducing tobacco use and in removing the negative influence of tobacco products in their communities, particularly for youth. For example, when responding to the policy to *ban the sale of flavored tobacco products* one woman shared: “Everyone has a favorite flavor of something they like but not being able to access this will make someone go nuts. It will reduce a lot of people from doing it.” For members of the Hispanic community, removing flavored tobacco products from the shelves was a perceived benefit of the Tobacco Endgame that would immediately improve public health within their community.

The second theme that emerged was policy suggestions. This was the only community that consistently embraced the potential for community members to influence policy language and type. For example, when responding to the request for *any other thoughts*, a female participant shared the following:

I personally think that it needs to bring more awareness to people and educate on the reasons why this Tobacco Endgame movement is very important. All the policies she just mentioned are great, but I feel like if I understood why something needed to be stopped, it would help me and a wide range of people. I also think that increasing the tax. If the price is higher and a lot of people can no longer afford it then it is going to decrease the demand. I think the price would only reduce a few range of people but maybe if it was a crime. if people were afraid of maybe being arrested. That would help a lot.

This is especially important as the inclusion of community members is outlined as a priority with the Endgame initiative. This finding suggests that Hispanic community members will prioritize participating in community-specific policy planning for the Tobacco Endgame initiative.

#### Unique themes among the AAPI community

Two unique themes emerged from focus groups with members of the AAPI community. First, similar to participants from the African American community, there was a consideration of the implications for businesses and local entrepreneurs. The perspective among these community members, however, was more positive, centering around revenue gain, ability to create work, and positive financial impact. For instance, in responding to the policy to *promote the proper cleaning of homes*,* vehicles*,* and other material goods*, a female participant shared the following perspective regarding how jobs could be created:

Definitely it will make selling and buying homes difficult, but at the same time proper. You know the real estate will have to come. Industry will have to come up with more rules. So it will affect the real estate industry. And I think the cleaners will go high in demand, and high in business. So definitely, you know, we will be generating more employment and more deep cleaning of the houses, which anyways is good for infectious diseases. So, I really think overall this is an amazing policy to implement.

The perceived economic benefits shared by AAPI participants provide a possible framework for Tobacco Endgame communication efforts to highlight various economic benefits that could follow the implementation of Tobacco Endgame policies.

Second, there was also a real concern about Tobacco Endgame policies infringing on the personal rights of smokers, specifically. For example, when responding to the policy to *expand smokefree policies in California*, one male participant shared:

I think sometimes it’ll add fuel to the very small minute group to just raise hell, you know, and bring the whole civil rights, you know, like human rights. And you know what; how far is Government gonna go? That’s the spin that’s usually put on it.

The perceived barrier of personal rights to smoke was echoed across all sessions, and suggests a dedicated need to remind communities that the opportunity to smoke is not a constitutional right .

#### Unique themes among the LGBTQ + Community

Finally, during focus groups with members of the LGBTQ + community, three unique themes emerged. Interestingly, all three of these were also present in another community group. Given that these sessions were racially and ethnically diverse, these similarities are not entirely surprising. First, like members of the African American community, the potential of the Tobacco Endgame policies to protect the environment was a clear priority. For example, in responding to the policy to *end the sale of tobacco products that contribute to environmental waste*, a female participant said:

Smoking is bad in general and it is very harmful for the environment. The amount of work that we have to do to get it out of our system and then out of our streets and then it comes back in the system in our water. I just hate cigarette butts all over the place.

The outdoor environment was perceived as an essential benefit of Tobacco Endgame policies, one that should be leveraged in communication efforts.

Second, and similar to participants in the sessions with AAPI community members, there were perceived economic benefits that could follow the implementation of a Tobacco Endgame policy. For example, a male participant responding to policies seeking to *promote the proper cleaning of homes*,* vehicles*,* and other material goods* said,

I think it would be a great incentive to get landlords to do a policy that my building is smokefree because they know it’s gonna be harder to rent out their units if their unit is listed on a list of units that have smokers in it. They want to be on the good list of like, you know, this is a clean apartment that everybody’s gonna want to live in. And so I think yeah, it’s gonna be effective in getting landlords to make that decision to have their building go smoke free because it’s gonna have a financial burden on them.

Emphasizing the potential for Tobacco Endgame policies to provide economic benefits is another way to reduce concerns over post-implementation challenges that communities might face.

Finally, similar to the AAPI sessions, concerns for individual rights emerged. However, unlike the AAPI sessions which were predominantly concerned for the individual rights of people who smoke, there was a perspective among the LGBTQ + community members that these policies were beneficial because they more adequately protected the personal rights of people who do not smoke. For example, when responding to the policy to *expand smokefree policies in California*, one person shared the following:

As a member of the LGBTQ community you know, we know, that LGBTQ bars are spaces that are traditionally safe spaces where people can go, and not worry about—you know—about the outside world and the rejection and homophobia, etc. And in these spaces, unfortunately, including the outdoor patio spaces, can often be smoking spaces, and unless there’s a specific city or county policy against it, you know, these should really be spaces, for you know everybody to enjoy fresh air and not have to breathe toxic smoke. And that’s also true, not just of customers in these spaces, but also staff workers in these spaces, bartenders and barback people who have to breathe this toxic air when they’re working. And nobody should have to risk their, risk their health rather to keep their job.

Emphasizing the personal rights of people who do not smoke to breathe clean air and live tobacco-free is an important policy benefit that Tobacco Endgame planners should consider leveraging in their communication.

In sum, unique themes emerged within each of the four community groups. The themes reflect community perceptions of both barriers and benefits of the various Tobacco Endgame policies. Together, the themes highlight the various ways in which the priority populations that have historically experienced disproportionate tobacco industry-targeting perceive the Tobacco Endgame policies.

## Discussion

The purpose of this study was to explore how communities targeted by the tobacco industry, notably racial and ethnically underserved minorities and members of the LGBTQ + community, perceive California’s Tobacco Endgame policy initiatives. Analyses revealed some similarities in how minority and underserved communities perceive Tobacco Endgame policies and priorities at the same time revealed each community’s unique perceived barriers and benefits of Tobacco Endgame policies. In addition to identified themes, the data also provides opportunities to correct misperceptions and misinformation. The findings provide critical messaging and policy implementation strategies that can aid equitably minded global Tobacco Endgame efforts.

First, support for the general idea of the Tobacco Endgame was consistently present. All community groups centered the role of health, addiction, post-implementation challenges, and children and youth within the discussion. Further, there was generally a positive perception of the various policies across all four communities. This suggests that ongoing statewide tobacco education efforts are successfully facilitating a level of Tobacco Endgame readiness, preparing communities to transition to tobacco-free environments. Efforts to remind Californians about Tobacco Endgame benefits for health and youth will likely garner further endorsement. Similarly, efforts to acknowledge how the policies will support those with a nicotine addiction and to clearly outline post-implementation procedures have the potential to reduce reactance and uncertainty. Given that these benefits and barriers were present for all four participating communities, this messaging could be effective in a state-wide campaign.

Second, regarding perceived barriers and benefits of the Tobacco Endgame, there are many unique opportunities to leverage community-specific perceived benefits and reduce community-specific perceived barriers. For California communities with large majorities of African American or LGBTQ + residents, highlighting how Tobacco Endgame policies help protect the environment will bolster support for policy initiatives. The fact that the Tobacco Endgame seeks to address issues of environmental justice [7] will be persuasive messaging for these two communities, in particular.

Further, efforts to emphasize positive impacts of Tobacco Endgame policies on the economy and reduce fears over the potential creation of black markets will reduce potential reactance to policy initiatives. For California communities with large majorities of AAPI and Hispanic residents, highlighting how Tobacco Endgame policies promote local businesses will likely reinforce support. This could include emphasizing how policies create an opportunity to sell healthier products as well as create an opportunity for new jobs to enforce tobacco control policies or to remove tobacco pollution, both indoors and outdoors. On social media, both anti- and pro-tobacco posts frequently focus on the economic impact [26]. Tobacco Endgame campaign strategies would benefit from actively being engaged in that debate and directly countering the pro-tobacco arguments.

Smoking is not a constitutional right [27]. The right to breathe clean air, however, has been argued as a clear human right [28, 29]. For California communities with large majorities of AAPI residents, directly tackling the misperception that people have a right to smoke will likely reduce reactance. Similarly, for California communities with large majorities of LGBTQ + residents, reminding people that everyone has a right to breathe clean air will likely promote support for Tobacco Endgame policies. Given the success of a communication campaign using the slogan *Let’s Clear the Air* to increase compliance with a university tobacco-free policy [30], such clean air-focused messaging could simultaneously highlight the personal rights as well as environmental benefits of Tobacco Endgame policies.

For California’s Hispanic communities, emphasizing the roll of Tobacco Endgame policies in eliminating the sale of flavored tobacco products will likely garner support for policy initiatives. This is especially important as adolescent vaping has been shown to be positively associated with the availability of flavored products [31–34]. As both anti- and pro-tobacco posts on social media frequently make posts regarding flavors [26], Tobacco Endgame campaign efforts should seek to be at the forefront of this ongoing conversation. However, before any education or communication campaign efforts are implemented, Hispanic community members, in particular, should be consulted and included in the policy planning and outreach. Considering the present Tobacco Endgame platform calls for stakeholder involvement throughout all stages of policy development and implementation [7], the findings here suggest that time and financial investment into such involvement opportunities for political advocacy groups representing community members will not be wasted, particularly among California’s Hispanic community.

Given the racism and discrimination that has historically plagued—and continues to disproportionately plague—the U.S. African American community [35], it is not surprising that concerns over increased criminalization among African American residents emerged during focus group discussions. Although presently outlined policies in California’s Tobacco Endgame initiative do not intend to criminalize individuals for smoking, that doesn’t mean that enforcement officials won’t use the policy language as a cover to harass and discriminate against African Americans seen smoking. The reality that some form of criminalization is being considered, in this case criminalization of those who sell tobacco products, is understandably worrisome for this community. Given the historical traumas experienced by African Americans in the U.S., there is an ethical and moral imperative for policy planners to make all efforts to reduce any risk of criminalization of users. Although the present Tobacco Endgame platform calls for prioritizing the use of treatment programs and cessation resources over traditional criminal justice approaches [7], outreach and education efforts attempting to assure African American communities that these policies won’t lead to more arrests will likely fall flat against the historical backdrop of messages being empty in practice. Instead, crafting policy language to legally prevent discriminatory criminalization will, over time, speak for itself. Most importantly, the inclusion of political advocacy groups representing members of the of African American community while crafting policy language is the best strategy for working to reduce the perceived and actual risks for this community.

Overall, implications of the findings provide community-specific messaging strategies for California’s Tobacco Endgame activists. Anti-tobacco message fatigue is on the rise among many community groups [25]. This points to a need for community-informed and evidence-based tobacco prevention messages that will break through highly saturated media environments to both reach and resonate with the target audience. The need for these findings responds to the present Tobacco Endgame platform goal of developing educational messages to reach priority populations [7]. Targeted efforts are essential for enhancing policy readiness within each community. All across the world, numerous qualitative investigations surrounding tobacco control have demonstrated the ability of community-level insights to provide a framework to guide messaging needs among target communities that contribute to reduced public reactance of tobacco-related policies [36–38]. Through leveraging and educating on the themes found here, community readiness for Tobacco Endgame policies can be bolstered and reactance can be reduced. These efforts will increase community preparedness for implementing policies that remove the influence of Big Tobacco. Although these findings reflect the perspectives of California residents, they could hold true as national efforts grow to implement the Tobacco Endgame. Regardless, they demonstrate the need for other regions to include the perspective of racial and ethnically underserved minorities and members of the LGBTQ + community in identifying effective tobacco prevention priorities and strategies.

### Limitations & future research

There are a few limitations of the study that need to be acknowledged. First, the sample represented limited geographic regions from across California with most participants from Southern California. Broader representation from across the state would enhance participant representation. Similarly, the sample included only four underserved and minority communities. These communities reflect some, but not all, of the priority populations suffering disproportionate tobacco-related burdens after decades of targeting by the tobacco industry. Gathering perspectives from other communities would enhance the ability to support all of California’s diverse communities. Finally, the number of participants in each session varied from group-to-group; more similar session sizes would have been preferred for consistency in participant experience.

The findings provide important directions for future research. First, public health practitioners should use the findings here to build readiness and support for California’s Tobacco Endgame via state-wide and community-targeted educational campaigns. Second, researchers should explore the extent to which these community perceptions, including benefits and barriers, hold true outside of southern California residents. As the Tobacco Endgame initiative grows globally, such information is important for preparing communities for a tobacco-free world.

## Conclusion

A cohesive and effective messaging strategy will be essential to ensure California achieves the Tobacco Endgame policy goals. Findings provide important direction for California’s advocates seeking to achieve these by 2035. The reported community-specific benefits and barriers highlight the information needs that can be addressed by communication and education outreach. Such focused efforts are essential for enhancing policy readiness within each community, reducing the potential for reactance and resistance. Further, universal experiences of concerns for health, children, people who do not smoke, and enforcement challenges provide recommendations for statewide prevention messaging likely to resonate broadly.

## Data Availability

The datasets generated and analyzed during the current study are not publicly available due to the confidential nature of the statements, but are anonymously available from the corresponding author on reasonable request.
